# Study of Alternative GPS Network Meteorological Sensors in Taiwan: Case Studies of the Plum Rains and Typhoon Sinlaku

**DOI:** 10.3390/s90605001

**Published:** 2009-06-24

**Authors:** Kai-Wei Chiang, Wei-Chih Peng, Yen-Hua Yeh, Kwo-Hwa Chen

**Affiliations:** 1 Department of Geomatics, National Cheng-Kung University / No.1, Ta-Hsueh Road, Tainan 701, Taiwan; E-Mails: kwchiang@mail.ncku.edu.tw (K.W.C.); p6697404@mail.ncku.edu.tw (W.C.P.); yenhua0307@gmail.com (Y.H.Y.); 2 Department of Real Estate and Built Environment, National Taipei University / No. 67, Section 3, Ming-Sheng East Road, Taipei 104, Taiwan

**Keywords:** precise point positioning (PPP), zenith tropospheric delay (ZTD), meteorological sensor

## Abstract

Plum rains and typhoons are important weather systems in the Taiwan region. They can cause huge economic losses, but they are also considered as important water resources as they strike Taiwan annually and fill the reservoirs around the island. There are many meteorological sensors available for investigating the characteristics of weather and climate systems. Recently, the use of GPS as an alternative meteorological sensor has become popular due to the catastrophic impact of global climate change. GPS provides meteorological parameters mainly from the atmosphere. Precise Point Positioning (PPP) is a proven algorithm that has attracted attention in GPS related studies. This study uses GPS measurements collected at more than fifty reference stations of the e-GPS network in Taiwan. The first data set was collected from June 1st 2008 to June 7th 2008, which corresponds to the middle of the plum rain season in Taiwan. The second data set was collected from September 11th to September 17th 2008 during the landfall of typhoon Sinlaku. The data processing strategy is to process the measurements collected at the reference stations of the e-GPS network using the PPP technique to estimate the zenith tropospheric delay (ZTD) values of the sites; thus, the correlations between the ZTD values and the variation of rainfall during the plum rains and typhoon are analyzed. In addition, several characteristics of the meteorological events are identified using spatial and temporal analyses of the ZTD values estimated with the GPS network PPP technique.

## 1. Introduction

**Figure 1.**
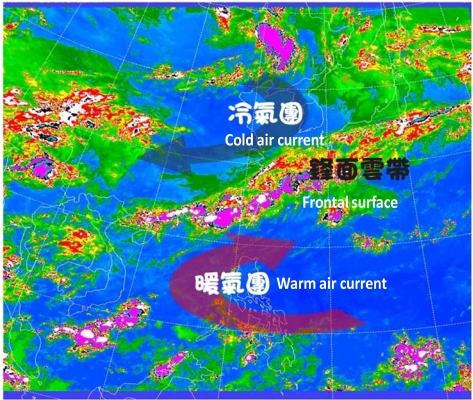
Sketch map of plum rains. Courtesy of the Central Weather Bureau (CWB), Taiwan.

Plum rains are the unique weather and climate phenomenon that take place annually from May to June in eastern Asia, including the Taiwan region and coastal China. In addition to typhoons, plum rains can cause a lot of damage in Taiwan every year as they produce a large amount of rainfall over a very short period of time. From spring to summer, the northeastern monsoon and the southwestern monsoon bring cold and warm air currents, respectively. When the air currents merge, they become a frontal surface, as shown in Figure 1.

**Figure 2.**
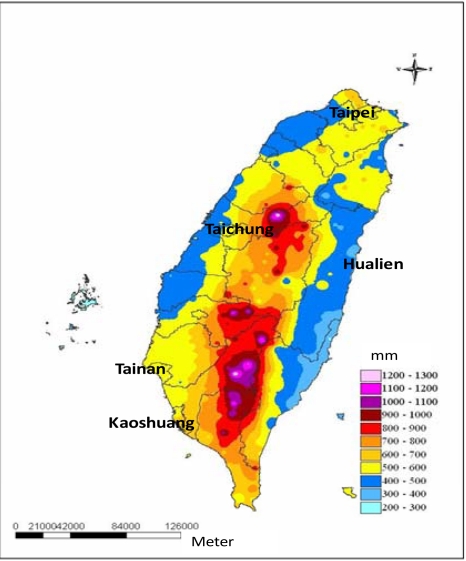
Average accumulated rainfall from 1992 to 2006 during the plum rain season (Courtesy of CWB, Taiwan).

Because the strengths of the air currents are similar, they often form a stationary front. This front moves slowly from south to north, bringing plentiful rainfall. The rainfall in the southwest of Taiwan is usually much higher than that in the northeast of Taiwan due to the southwestern air current. As the frontal surface gradually moves north, the rainfall in the northern part of this region stops and the rainfall in the southern part remains. Figure 2 shows the average accumulated rainfall from 1992 to 2006 during the plum rain season (May and June).

Typhoons are the most severe weather system in Taiwan. Four or five typhoons affect Taiwan every year, bringing destructive winds and plentiful rainfall. They pose a danger to agriculture, industry, and human life. For example, the economic losses due to typhoons can be up to 20 billion NT dollars and that due to plum rains can be up to 1.7 billion NT dollars [1].

**Figure 3.**
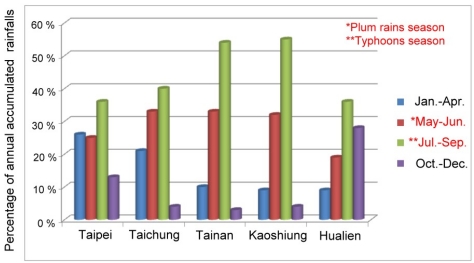
Annual distribution of accumulated rainfall (Courtesy of CWB, Taiwan).

Despite their devastating effect, typhoons and plum rains together account for more than 50% of the annual accumulated rainfall, as shown in Figure 3. They are thus an important water resources which fill reservoirs around the island. Consequently, studies that develop effective monitoring approaches or tools to monitor these events are important because they can be used to prevent the loss of human life and economic activity.

## 2. Problem Statements

There are many meteorological sensors available for studying the patterns and characteristics of meteorological events. Recently, many studies have used GPS as an alternative meteorological sensor. Distributed GPS stations around the earth can serve as a near real-time meteorological sensor network that can be used to detect potential threats. Continuous and well-distributed measurements of water vapor are of great interest for numerical weather forecasts, climate research, and atmospheric studies.

GPS signals going through the atmosphere get delayed by ionosphere and troposphere refractions. The refraction due to the ionosphere (the ionosphere is a dispersive medium) can be easily removed by linear combinations of dual frequency measurements because the ionospheric delay depends on the signal frequency [2].

When processing single frequency observations, the observations have to be corrected for ionospheric effects. Available empirical models include the broadcast Klobuchar ionospheric model, the post fit Klobuchar ionospheric model estimated by the Center for Orbit Determination in Europe (CODE), and the Global Ionospheric Maps available from International GNSS Service (IGS), respectively. The positional accuracy is generally not better than sub-meter when processing observations from single frequency receivers because of ionospheric effects.

The tropospheric delay cannot be removed using dual frequency measurements because in the GPS signal frequency band the troposphere is non-dispersive. The tropospheric delay is composed of two parts: a hydrostatic delay term that depends on atmospheric pressure and temperature, and a wet delay term that depends on the partial pressure of water vapor and temperature. While the hydrostatic delay can be accurately modeled using surface pressure measurements, the wet component is difficult to model because of its large spatial and temporal variability. Because meteorological values are insufficient to account for the wet path delay, wet tropospheric delays are usually estimated as an unknown parameter.

Reference [3] presented the idea of using GPS for measuring water vapor. This discussion shows a technical challenge using GPS to measure atmospheric water vapor. The total delay of the radio signals between a GPS satellite and a ground GPS receiver is essentially dependent on the total atmospheric mass, *i.e.*, the pressure at the surface, and the columnar atmospheric moisture content.

Consequently, the primary meteorological measurements produced by GPS are tropospheric path delays at the zenith. The ZTD is estimated from the line of sight tropospheric delays corresponding to the GPS satellites in view. Each of these tropospheric delays is mapped to the zenith using an elevation dependent mapping function. The zenith tropospheric path delay of GPS signals can provide essential information on the long-term climate change of a given area of study.

Current carrier phase based GPS kinematic positioning systems are primarily based on a double differencing data processing approach which can provide centimeter to decimeter positional accuracy in real-time. Since the reduction of common errors depends on the inter-station baseline lengths, the base and rover station separation must be short, typically about 20 kilometers. The need for a base station increases the cost of equipment and labor and introduces inconsistency [4].

The availability of precise GPS satellite orbit and clock products provided by IGS has enabled the development of a novel positioning methodology known as PPP [5]. Based on the processing of pseudorange and carrier phase observations from a single GPS receiver, this approach effectively eliminates the inter-limitation introduced by differential GPS (DGPS) processing as no base station is necessary. Therefore, PPP offers an alternative to DGPS that is simpler than and almost as accurate as DGPS [6].

Users can directly use IGS products to obtain highly accurate frames of International Terrestrial Reference Frame (ITRF) using the PPP technique. This decreases costs because PPP needs only one receiver to perform high positioning accuracy, therefore, it is widely used in studies of the atmosphere, meteorology, and tides [7].

The present study investigates the applicability of using GPS network processed in PPP mode as meteorological sensors to monitor the characteristics of plum rains and typhoons in the Taiwan region.

## 3. PPP Based Tropospheric Delay Estimation

As mentioned in the previous section, the zenith tropospheric delay is often composed of two parts: a hydrostatic delay term and a wet delay term. About 85-90% of the tropospheric delay comes from the dry component which can be accurately modeled with a precision approaching 1% when the pressure is known (to mm accuracy) using most available troposphere modeling methods. The wet component, however, is more difficult to model because of its large spatial and temporal variability; an error of 15-20% is common.

**Figure 4.**
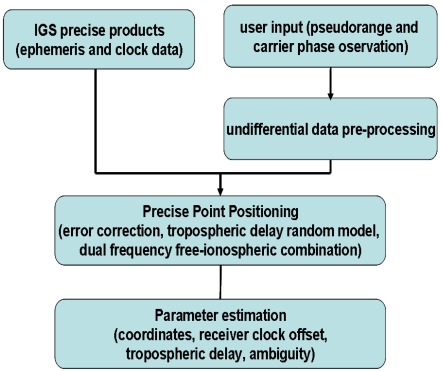
PPP data processing strategy.

Since the wet zenith troposphere delay residual is still significant for cm level positioning after a tropospheric correction model is used, it should be estimated as an unknown parameter along with other parameters such as the coordinates, the receiver clock offset, and the ambiguity. Figure 4 shows the data processing strategy of PPP. The details mathematical model of PPP can be found at [7]. To obtain an accurate zenith tropospheric wet delay estimate, the total tropospheric delay can be modeled as [8]:

(1) $$d_{\text{trop}}=m_{h}\left(e\right)\cdot D_{hz}+m_{w}\left(e\right)\cdot D_{wz}+m_{g\left(e\right)}\cdot \left[G_{N}\cdot \cos\left(a\right)+G_{E}\cdot \sin\left(a\right)\right]$$

where:

*D_hz_* the zenith hydrostatic delay
*D_wz_* the zenith wet delay
*G_N_ G_E_* the horizontal delay gradient in the north and east directions
*m_h_*(*e*) the hydrostatic mapping function
*m_w_*(*e*) the wet mapping function
*m_g_*(*e*) the gradient mapping function
*a e* the azimuth and elevation angles

The estimation of tropospheric gradients is included to take into account the horizontal atmospheric variations. Neill Mapping Functions (NMFs) are applied for the hydrostatic and wet delays in this study. The following function was applied for gradient mapping [9]:

(2) $$m_{g}\left(e\right)=\frac{1}{\sin\left(e\right)\cdot \tan\left(e\right)+0.0032}$$

The current DGPS based wet zenith troposphere delay estimate approach can only provide relative values between two reference stations and it requires a Water Vapor Radiometer (WVR) to be set up at one of those stations to provide proper calibration to obtain absolute zenith troposphere delay at the other site. In practice, the WVR is too expensive and fragile to operate during the visitation of typhoons as well as thunderstorms. Thus, the applicability of a conventional DGPS/WVR based approach to monitor the extreme weather events is rather limited. On the other hand, PPP can provide the direct estimate of absolute wet zenith troposphere delay by using a dual frequency GPS receiver with meteorological sensors that can provide pressure, temperature and humidity. It does not require a WVR to perform calibration and its data processing scheme is straight forward comparing to DGPS data processing. Therefore, when processing massive amount of measurements provided by a GPS network, PPP based approach is a cost effective option. In addition, [10] indicated the difference between the wet zenith troposphere delay the provided PPP and WVR was in the scope of several millimeters that can be considered acceptable for meteorological study.

## 4. The e-GPS Network

**Figure 5.**
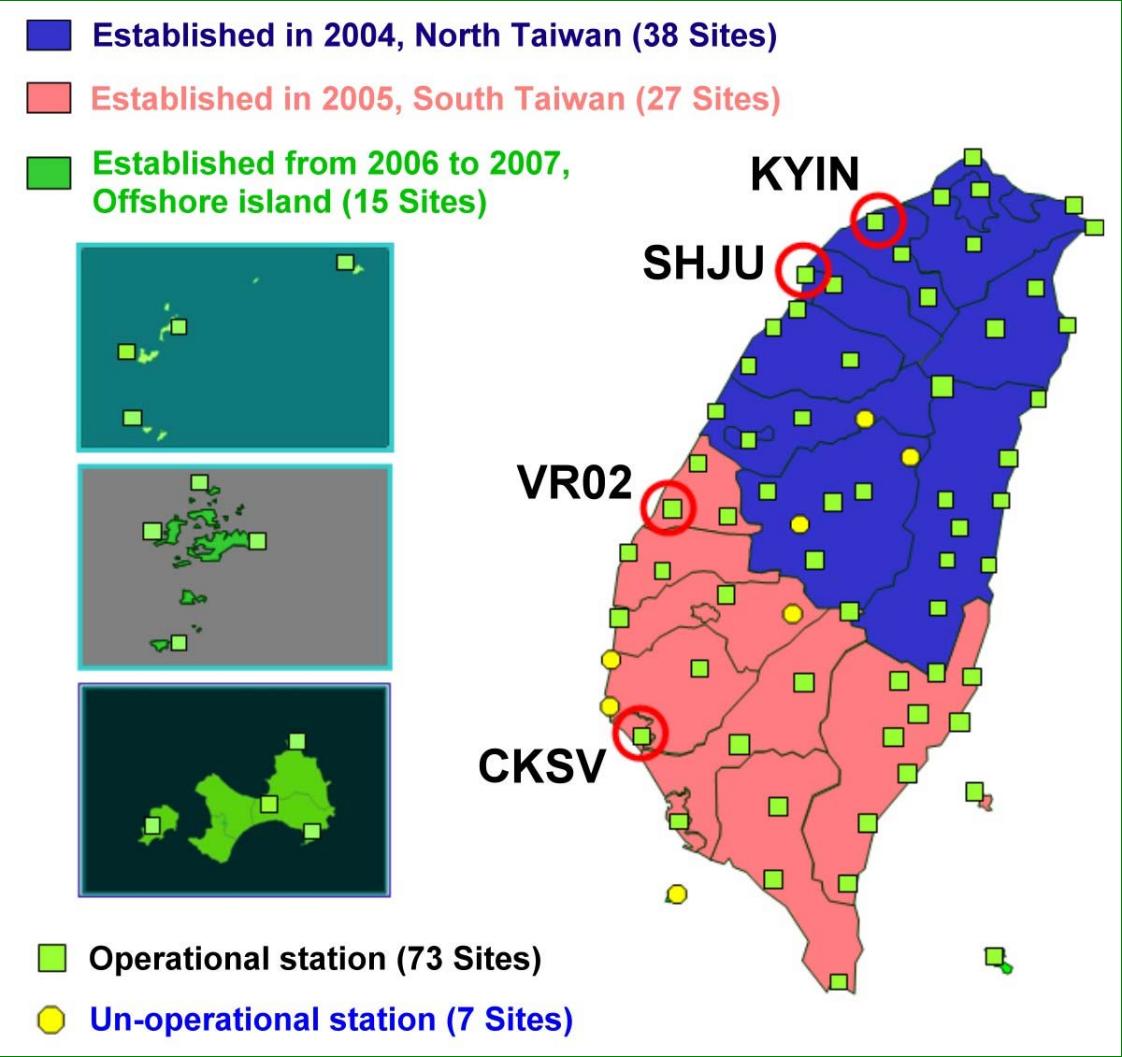
Distribution of e-GPS observation stations (Courtesy of NLSC, Taiwan).

The electronic-global satellite real-time kinematic positioning system (e-GPS) is managed by the National Land Survey and Mapping Center (NLSC), Ministry of the Interior, Executive Yuan of ROC (Taiwan). The e-GPS network uses continuous satellite observations and processes them continually. Users need to receive more than four GPS satellite signals to obtain high accuracy positioning coordinates in a short period of time. Figure 5 shows the distribution of the entire e-GPS network, which consists of approximately eighty operational stations located around the main island and on most of the offshore islands.

Most on-site GPS receivers of the e-GPS network operate 24 hours a day, even under severe weather conditions, such as typhoons and plum rains. Therefore, this well distributed e-GPS network processed in PPP mode can be considered as a supplemental meteorological sensor to study the patterns of typhoons and plum rains.

## 5. Results and Discussions

**Figure 6.**
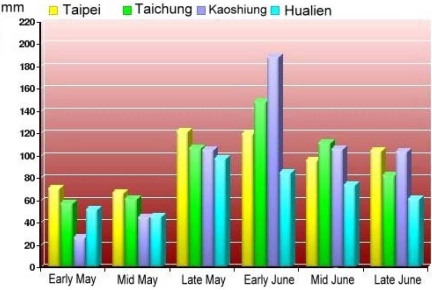
Geographical distributions of the average accumulated rainfalls (Courtesy of CWB, Taiwan).

As mentioned previously, plum rains often occur from the middle of May to the middle of June. Therefore, the data set used to investigate the characteristics of plum rains was collected from June 1st 2008 to June 7th 2008. This time frame is right in the middle of plum rain season in Taiwan. Figure 6 shows the average accumulated rainfalls during plum rain season for major cities on the island (see Figure 2).

**Figure 7.**
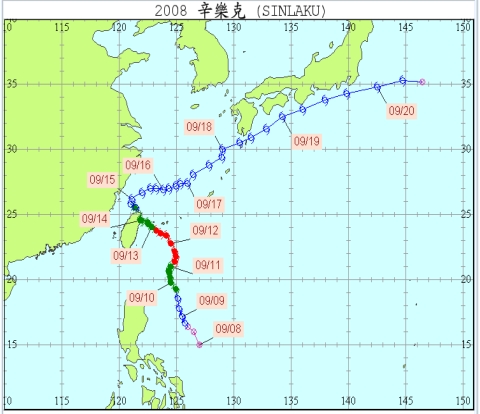
Path of Typhoon Sinlaku (Courtesy of CWB, Taiwan).

Figure 7 shows the path of typhoon Sinlaku that made landfall from the western Pacific. A sea warning for the typhoon was issued at 8:30 AM, on September 11th, 2008, and the land warning was issued at 2:30 PM, on September 16th, 2008. The data set used to study the characteristics of Typhoon Sinlaku was collected from September 11th to September 17th.

**Figure 8.**
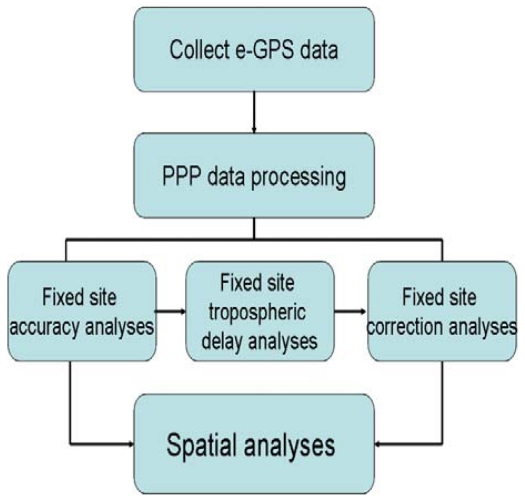
GPS data processing scheme.

Figure 8 shows the data processing scheme used in this study. The measurements collected at most of the operable e-GPS observation stations were processed with the PPP technique to obtain tropospheric delay corrections and the site coordinates. The spatial and temporal variations of ZTD values estimated from the e-GPS network were analyzed with the movement of typhoon Sinlaku and plum rains.

The velocity of Typhoon Sinlaku was about 50 meters per second; the radius of the typhoon was approximately 250 kilometers. The typhoon made landfall in the northeast coast near Yilan at 1:40 AM, on September 14th, 2008. It left the island near Taipei at 8:00 AM, on September 15th, 2008. The ZTD values estimated by PPP and accumulated rainfall collected by the observation posts of CWB were normalized. The normalized algorithm of ZTD (or accumulated rainfall) is interpreted as Equation (3):

(3) $$NL_{i}=\frac{X_{i}-X_{\min}}{X_{\max}-X_{\min}}$$

where *NL_i_* denotes the normalized value of ZTD (or accumulated rainfall); *X_i_* is the realistic ZTD value estimated by PPP (or the realistic accumulated rainfall at the collected time); *X_min_* and *X_max_* represent the minimum and maximum value of the realistic ZTD value (or the realistic accumulated rainfall) during the collected period, namely, Jun-01-2008 to Jun-07-2008, respectively. More than fifty e-GPS sites were used in this study; the number of CWB posts was twenty five. The density of the e-GPS network based monitoring approach is twice that of conventional meteorological sensors.

### 5.1. Spatial Correspondence between ZTDs and Rainfalls during Typhoon Sinlaku Influencing Period

**Figure 9.**
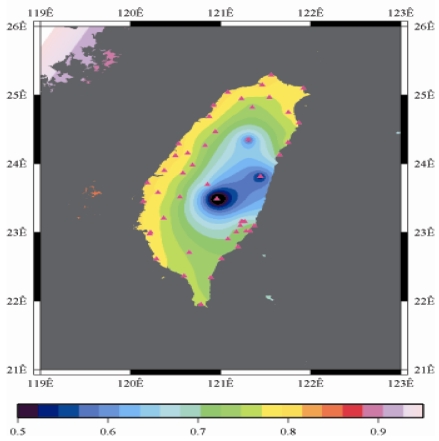
Normalized ZTD values around the island before the typhoon struck Taiwan (September 13th, 2008, 3:00 PM).

**Figure 10.**
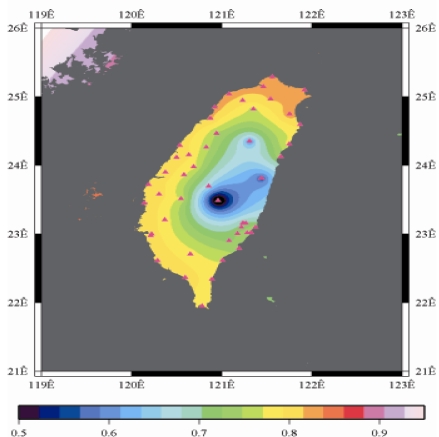
Normalized ZTD values when the typhoon started to strike Taiwan (September 13th, 2008, 11:00 PM).

**Figure 11.**
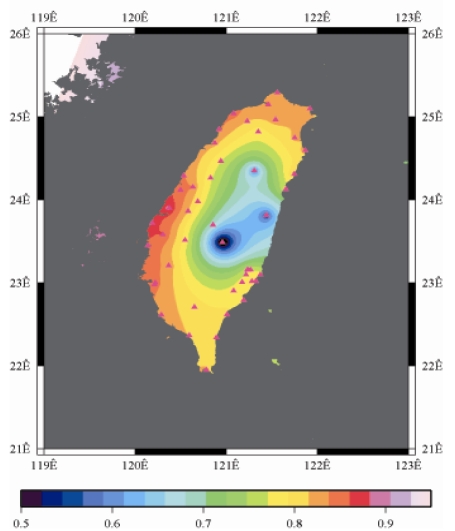
Normalized ZTD values around the island when the typhoon was striking Taiwan (September 14th, 2008, 1:00 PM).

**Figure 12.**
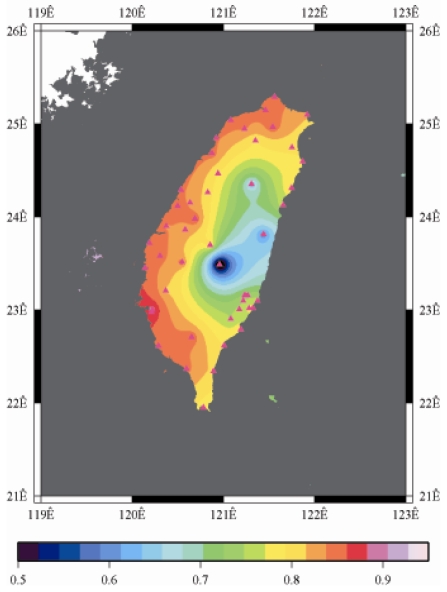
Normalized ZTD values around the island after the typhoon struck Taiwan (September 15th, 2008, 9:00 AM).

Figures 9 to 12 show the spatial distribution of normalized ZTD values before and after the typhoon struck the island, respectively. The normalized ZTD values estimated at e-GPS sites in the Central Mountain area are always low because of the altitude. On average, the altitude exceeds two thousand meters above sea level.

**Figure 13.**
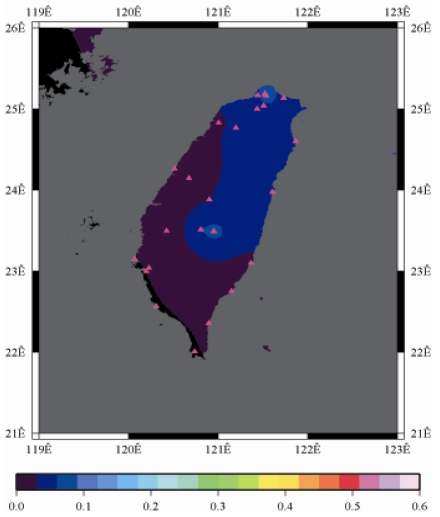
Normalized rainfall values around the island before the typhoon struck Taiwan (September 13th, 2008, 3:00 PM).

**Figure 14.**
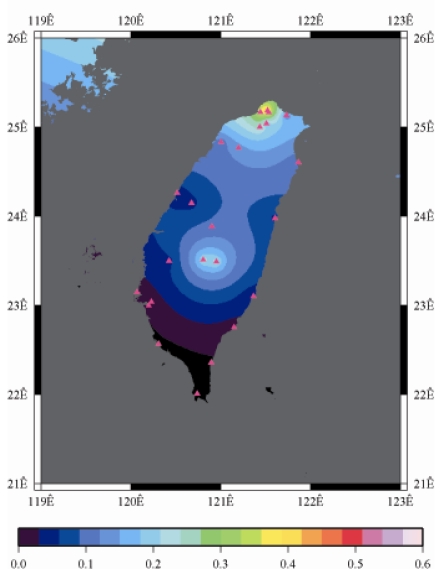
Normalized rainfall values around the island when the typhoon started to strike Taiwan (September 13th, 2008, 11:00 PM).

**Figure 15.**
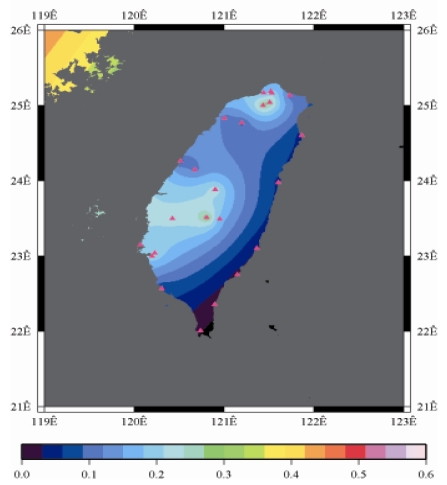
Normalized rainfall values around the island when the typhoon was striking Taiwan (September 14th, 2008, 1:00 PM).

**Figure 16.**
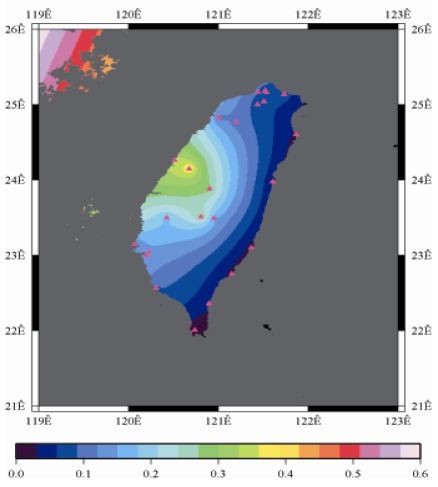
Normalized rainfall values around the island after the typhoon struck Taiwan (September 15th, 2008, 9:00 AM).

Figures 13 to 16 show the spatial distribution of normalized rainfall collected at the CWB meteorological posts during this period of time. The spatial distributions of normalized rainfall fit the normalized ZTD values closely. The geographic locations of e-GPS sites and CWB meteorological posts are shown in Figures 9 and 13, respectively. Since the spatial distribution of e-GPS sites is more complicated than that of CWB posts, e-GPS provides higher spatial resolution for monitoring meteorological events.

Before the typhoon made landfall, the normalized values of ZTD and rainfall were quite low, except for the north of the island, as shown in Figures 9 and 13, respectively. The northern part of the island was covered with minor rain activity during this period of time. As the typhoon approached the island, the rainfall gradually increased. The normalized values of ZTD and rainfall in northeastern Taiwan increased, as shown in Figures 10 and 14, respectively. When the typhoon made its landfall at northern Taiwan, the external monsoon of the typhoon went along with the southwestern monsoon and affected western Taiwan.

The rainfall in the western region increased significantly; the normalized values of ZTD and rainfall gradually increased, as shown in Figures 11 and 15, respectively. Terrestrial rainfall was produced by the typhoon entering the mountain area in northern Taiwan as the typhoon rotated counter–clockwise. Thus the north of Taiwan suffered the impact of terrestrial rainfall when the moist air was blocked by the Mountains when Typhoon made its landfall at the northeast of Taiwan.

Eventually, the entire western region was covered with heavy rain; the normalized values of ZTD and rainfall were remained high. The typhoon left Taiwan from the northwest. When typhoon left Taiwan completely, the exterior air current of typhoon with the strong southwestern monsoon made the central of Taiwan suffered serious terrestrial rainfall, as shown in Figures 12 and 16, respectively. Similarly, the phenomenon conform the terrestrial rainfall produced by the encounter of typhoon and Central Mountain as the typhoon rotated in counter clockwise direction thus the central part of Taiwan suffered the impact of terrestrial rainfall when the moist air was blocked by the Central Mountain.

### 5.2. Spatial Correspondence between ZTDs and Rainfalls during Plum Rains Period

**Figure 17.**
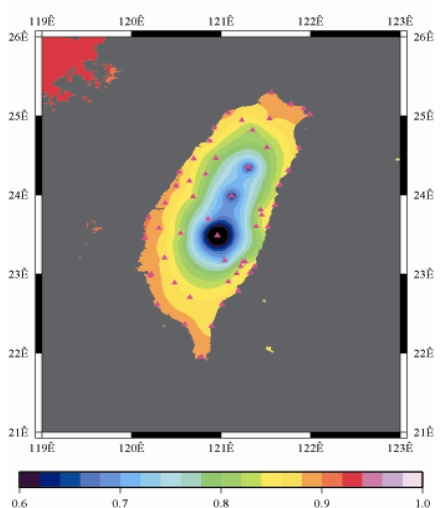
Normalized ZTD values around the island before plum rains (June 2nd, 2008, 5:00 AM).

**Figure 18.**
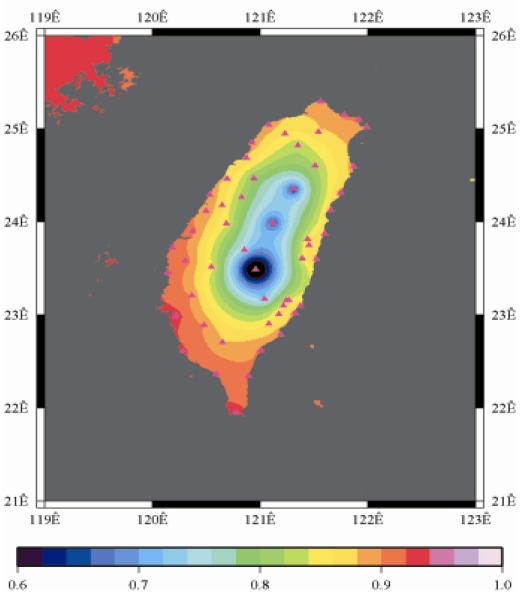
Normalized ZTD values around the island during plum rains (June 2nd, 2008, 1:00 PM).

**Figure 19.**
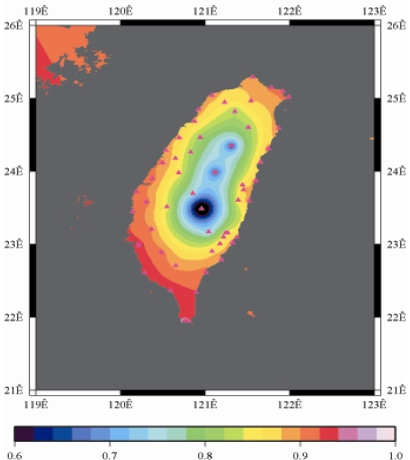
Normalized ZTD values around the island during plum rains (June 2nd, 2008, 5:00 PM).

**Figure 20.**
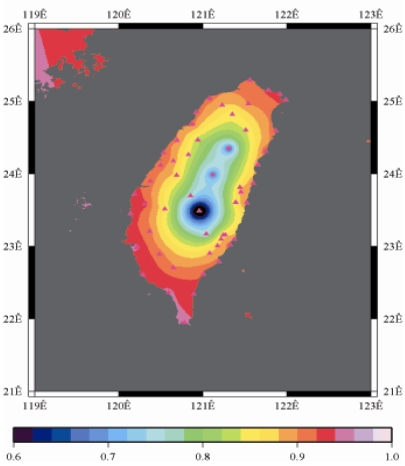
Normalized ZTD values around the island when the plum rains gradually moved north (June 3rd, 2008, 12:00 AM).

**Figure 21.**
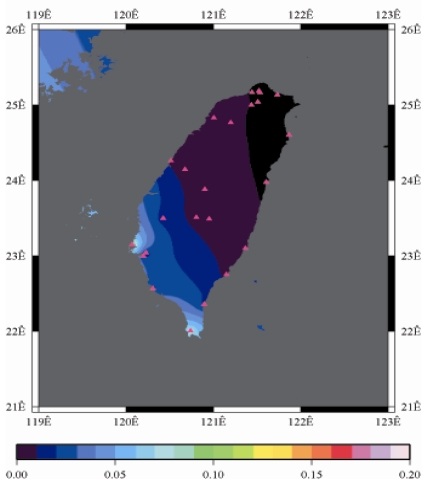
Normalized values of rainfall before plum rains (June 2nd, 2008, 5:00 AM).

**Figure 22.**
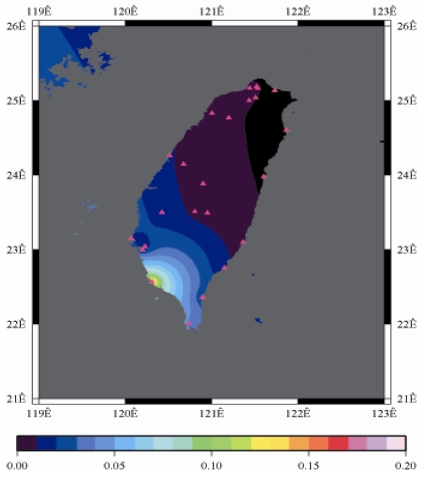
Normalized values of rainfall during plum rains (June 2nd, 2008, 1:00 PM).

**Figure 23.**
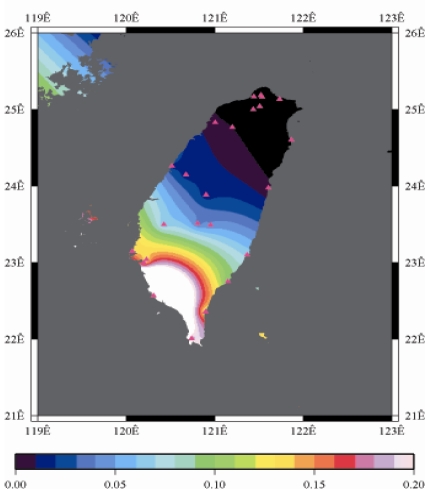
Normalized values of rainfall during plum rains (June 2nd, 2008, 5:00 PM).

**Figure 24.**
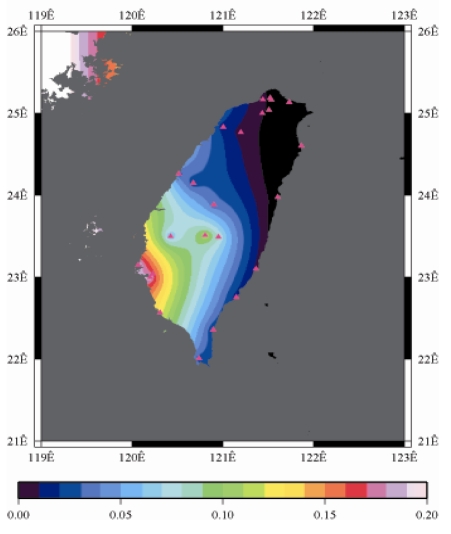
Normalized values of rainfall when the plum rains gradually moved north (June 3rd, 2008, 12:00 AM).

**Figure 25.**
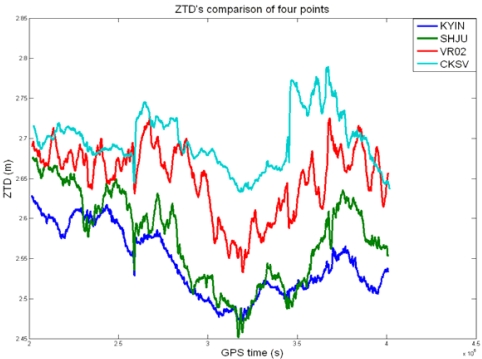
ZTD comparison of e-GPS stations in the north, northwest, southwest, and south during the plum rains.

**Figure 26.**
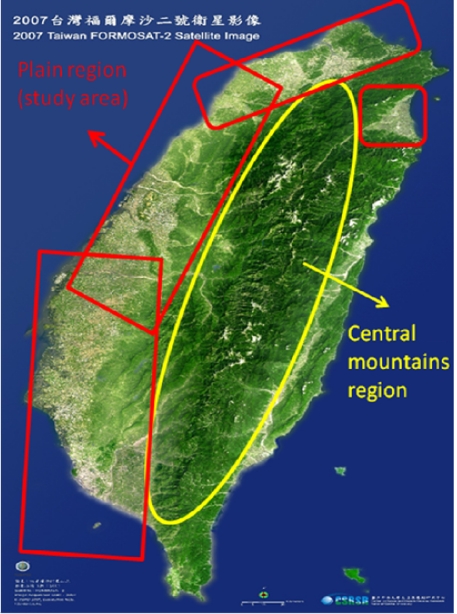
The terrain of Taiwan (FORMOSAT-2 Satellite Image, Courtesy of CSRSR, NCU, Taiwan).

The plum rain season often starts in May and ends in June, lasting approximately a month. The northeastern monsoon and southwestern monsoon cause a stationary front to remain in the northeastern region of the island. Although the strength of the northeastern monsoon is much stronger than that of the southwestern monsoon, it is usually blocked by the Central Mountains. Therefore, the stationary front gradually moves north, increasing rainfall in the north region. Figures 17 to 27 show the correlation between the normalized values of ZTD and rainfall during the plum rain season. As shown in the figures, the rainfall in southwestern Taiwan is usually much higher than that in northeastern Taiwan due to the southwestern air current. When the frontal surface gradually moves north, the rainfall in the northern part of this region stops but the rainfall in south part remains.

Generally speaking, the spatial distributions of normalized ZTD values estimated using PPP have similar characteristics to those of normalized values of rainfall recorded at CWB meteorological posts. Figure 25 shows that the rainfall in the south is much higher than that in the north. The rainfall collected at CWB posts near the e-GPS stations shows the same trend, as shown in Figure 3. The spatial distribution of the sites is shown in Figure 5. Figures 2, 20, and 23 show the same characteristics of plum rains. In other words, the proposed PPP can be used as effective sensors to monitor the characteristics of plum rains

As is well known, ZTD is altitude-correlated. The influence of site's altitude on ZTD should be removed before comparing with rainfall data by tropospheric correction model using the site's height information and the meteorological data, e.g. temperatures, humidity and pressure…etc. However, there were less than five e-GPS sites that had meteorological sensors onsite during the collected period thus it is not able to remove the influence of altitude in the mountain region without meteorological measurements with proper models. In fact, the impact of altitude on ZTDs in the mountain region are shown Figures 9 to 12 and 17 to 20 where the central mountain chains are darker-colored illustrating the smaller values comparing to the plain area. The terrain of Taiwan is shown in Figure 26. Therefore, the lack of meteorological measurements with proper models prevents us to look into the correlation between ZTDs and rainfall across the whole island, especially in the central mountain region (Figure 26).

Consequently, the aim of this paper is to limit the scope of interpretations and discussions of the relationship between ZTDs and accumulated rainfall in the plain area marked by red rectangular in Figure 26 because height variations in those regions are relatively smaller than in the central mountainous area. Therefore, the influence of altitude is so small that could be ignored in the west plain area of Taiwan as the purpose of this paper is to look into the spatial distributions between ZTDs and accumulated rainfall during typhoon and plum rains. On the other hand, the perceptible water vapour is definitely necessary if this study plan to look into the numerical relationships between ZTDs and accumulated rainfall during typhoon and plum rains. However, this requires meteorological measurements with proper models and it cannot be implemented with e-GPS infrastructures at the moment.

The well-distributed e-GPS network can be considered as supplemental meteorological sensors to monitor the patterns of typhoons and plum rains. With improved ultra-rapid products provided by IGS in the near future, or with real-time precise satellite orbit and clock products provided by other institutions, a near real-time PPP based meteorological sensors can provide useful information for developing early warning systems for meteorological events like typhoons, plum rains, and seasonal thunderstorms in this region.

## 6. Conclusions

This study proposed using ZTD values estimated from the well-distributed e-GPS network of NLSC and PPP technique to implement supplemental meteorological sensors to investigate the characteristics of typhoons and plum rains. The preliminary results show strong correlations between the variations of ZTD values estimated using PPP and rainfall recorded at meteorological posts around the island during plum rain season and the passage of typhoon Sinlaku.

Several characteristics of typhoons, including the terrestrial rainfall in northwestern and central Taiwan during the passage of typhoon Sinlaku, are identified using the spatial and temporal analyses of ZTD values estimated from PPP. The well-distributed e-GPS network combined with PPP can be considered as alternative meteorological sensors for monitoring the patterns of typhoons and plum rains.
